# Single nucleotide polymorphisms in the *CD40* gene associate with the disease susceptibility and severity in knee osteoarthritis in the Chinese Han population: a case-control study

**DOI:** 10.1186/s12891-017-1466-8

**Published:** 2017-03-21

**Authors:** Zhen-Han Deng, Ming-Hua Sun, Yu-Sheng Li, Wei Luo, Fang-Jie Zhang, Jian Tian, Ping Wu, Wen-Feng Xiao

**Affiliations:** 1 Department of Orthopaedics, Xiangya Hospital, Central South University, Changsha, 410008 China; 2 Department of Orthopaedics, Affiliated Hospital, Logistics University of Chinese People’s Armed Police Forces, Tianjin, 300162 China; 3 Department of Emergency Medicine, Xiangya Hospital, Central South University, Changsha, 410008 China

**Keywords:** Knee osteoarthritis, *CD40*, Single nucleotide polymorphism, Susceptibility, Severity, Rs4810485 G > T, Rs1883832 C > T

## Abstract

**Background:**

This study explored the association between single nucleotide polymorphisms (SNPs) in the *CD40* gene, rs4810485 G > T and rs1883832 C > T, as well as disease susceptibility and severity in knee osteoarthritis (KOA) in the Chinese Han population.

**Method:**

Peripheral venous blood was collected from 133 KOA patients (KOA group) and 143 healthy people (control group) from December 2012 to November 2013. The patients in the KOA group were classified into mild, moderate and severe groups according to disease severity. Polymerase chain reaction–restriction fragment length polymorphism (PCR-RFLP) was used to test the genotypes of all subjects. Binary logistic regression analyses were performed to analyze the risk factors for KOA.

**Results:**

The KOA group was significantly different from the control group in living environment (*P* < 0.05). The KOA group had a lower frequency of TT genotype and T allele distribution of rs4810485 G > T compared with the control group, and rs4810485 G > T TT genotype and T allele may associate with low incidence of KOA (all *P* < 0.05). Besides, T allele and mutant homozygous TT genotype of rs1883832 C > T increased the susceptibility to KOA. Genotype and allele distribution of rs4810485 G > T and rs1883832 C > T were significantly different among the mild, moderate and severe groups (*P* < 0.05). There were more patients with rs4810485 G > T GG genotype and rs1883832 C > T TT genotype in the severe group than other genotypes of these two SNPs. According to binary logistic regression analysis, rs4810485 G > T TT genotype could alleviate disease severity in KOA, rs1883832 C > T TT genotype increase the severity of KOA and living environment is an important external factor that affects KOA severity.

**Conclusions:**

These data provide evidences that rs4810485 G > T and rs1883832 C > T in the CD40 gene may be associated with disease susceptibility and severity in KOA.

**Electronic supplementary material:**

The online version of this article (doi:10.1186/s12891-017-1466-8) contains supplementary material, which is available to authorized users.

## Background

Knee osteoarthritis (KOA) is a degenerative disease with joint pain, stiffness, and function degeneration with a more prevalent incidence among middle- and old-aged groups [1, 2]. The clinical treatment for KOA patients usually combines drug therapy and non-drug therapy, among which the latter mainly includes exercise and acupuncture, and common KOA drugs include non-steroidal anti-inflammatory drugs, intra-articular glucocorticoid and intra-articular hyaluronic acid [3]. Although KOA is affected by many factors, the damage of meniscus occurred in the process of reconstruction surgery is a major cause of KOA [4]. According to statistics, patients with both anterior cruciate ligament (ACL) injury and meniscus injury have an incidence rate of KOA as high as 70% within 15 to 20 years after injury [5]. It is found that similar to osteoporosis and fracture; reduced cartilage thickness may also be one of the factors that predispose to KOA [6]. Due to the susceptibility and high incidence of KOA, it is of great importance to find early diagnosis markers of KOA to improve KOA treatment effect [2].

Cluster of Differentiation 40 (CD40) is a type I trans-membrane glycoprotein composed of 277 amino acids with a molecular weight of 40 ~ 50 kDa [7]. Human *CD40* gene is located at chromosome 20q11-13, containing eight introns and nine exons [8, 9]. Through interaction with its ligand CD40L, *CD40* plays an important role in cellular and humoral immunity, and it can activate inflammatory response in the human body and promote the progression of atherosclerosis [10, 11]. *CD40* rs1883832 C > T includes CC, CT, and TT genotypes with their promoters located at Kozak region, in which the occurring gene mutation greatly impacts the gene translation efficiency [12]. At present, a number of studies have found that single nucleotide polymorphisms (SNPs) at *CD40* gene promoter -1 is associated with numerous immuno-inflammatory diseases, such as Graves’ disease as well as acute coronary syndrome (ACS) [13, 14]. Although previously classified as a non-inflammatory arthritis, osteoarthritis (OA) has now been generally recognized as a inflammatory disease [15]. *CD40* rs1883832 was found associated with biopsy-proven giant cell arteritis (GCA) [16]. Moreover, the association of rs4810485 G > T with systemic lupus erythematosus (SLE) has been investigated by many researchers but without consistent results [17, 18]. Vazgiourakis found that rs4810485 G > T minor allele T is under-represented in SLE patients and correlates with reduced *CD40* expression [19]. Nevertheless, the literature investigating association between *CD40* rs4810485 G > T and rs1883832 C > T and KOA was limited in numbers. This paper intends to explore the relationship between SNPs in the *CD40* gene (rs4810485 G > T and rs1883832 C > T), disease susceptibility and severity in KOA, so as to find an effective target for early diagnosis and treatment of KOA.

## Methods

### Ethics statement

This study was approved by the Ethics Committee of Xiangya Hospital, Central South University and in accordance with the standards of the National Research Council. Informed consent was obtained from each patient prior to our study.

### Study subjects

A total of 133 patients diagnosed with KOA in Xiangya Hospital, Central South University from December 2012 to November 2013 were recruited as KOA group, (*n* = 133) comprised of 39 males and 94 females with a mean age of 58.24 ± 9.66 years. KOA diagnosis was in accordance with the KOA criteria laid down by Association of Rheumatology Health Professionals (ARHP) [20]. Exclusion criteria are as follows: joint diseases caused by other reasons, such as inflammatory arthritis, traumatic arthritis, suppurative arthritis, chronic inflammation, infectious diseases and tumor or skeletal dysplasia; patients with no less than 3 metacarpophalangeal joint involvements that are in grade 2–4 of Kellgren-Lawrence (KL) grading. Another 143 healthy individuals were included into the control group, containing 52 males and 91 females with a mean age of 59 (58.2 ± 6.7) years. Members in the control group underwent clinical examination, and X-ray confirmed that they did not have KOA or other arthritis. They also had no symptoms and signs of related joint diseases such as pain, swelling, tenderness and limited activity, or family history of KOA. All subjects had complete clinical data, including age, sex, body mass index (BMI), smoking and drinking status, heavy physical labor and living environment [21]. Smoking status was be divided into three categories: (1) never smoking; (2) used to smoke; (3) still smoking. People who are addicted to smoking were excluded from this study as subjects. In China, the light, medium and heavy drinking scales can be defined as 1.3 ~ 20 g, 20 ~ 50 g and > 50 g of daily alcohol intake. All patients in this study were light or occasional drinkers, indicating daily alcohol intake not exceeding 20 g and 2 ~ 3 times a week. For an 8 h work-day, if the average 8-h energy consumption is no less than 7310.2 J/person and working time ≥ 73%, namely, if the working time is more than 350 min, it can be defined as heavy labor. Living environment is closely related to human life, where light intensity and air humidity are two objective factors affecting living environment, according to which the research subjects can be divided into two types: (1) bright + dry type; (2) damp + dark type.

### Criteria for disease severity in KOA

In accordance with the severity grading of KOA clinical comprehensive index [22], six indicators (pain, swelling, walking difficulty, joint friction sound (sense), limitation of activity and X-ray change) were used to assess the KOA severity. According to the accumulative score of each indicator, mild means 6 points, moderate 7 to 11 points, and severe 12 to 18 points. In the KOA group, the mild group is comprised of 49 cases, moderate group 44 cases, and severe group 40 cases. Patients in the mild group exhibited no obvious symptoms; instead they usually felt joint stiffness, knee coldness or discomfort which could be slightly improved after activity. In the moderate group, patiens showed acute knee inflammation after violent activity, which was relieved by rest or symptomatic treatment. Knee ache and discomfort occurred when sitting up and symptoms were relieved after walking for a while. Patients in the severe group had unbearable knee pain, and had difficulty of going upstairs and downstairs, as well as squatting or standing. A long walk would cause swelling in the knee joint with some mucus. Moving their knees would cause a sound along with limitation of activity, joint deformity and even potential crippling.

### Polymerase chain reaction–restriction fragment length polymorphism (PCR-RFLP)

Based on the genomic data of Chinese Han population in HapMap, this study was conducted as follows: literature review was done before searching for Tag-SNP and FAST SNP [23] to find the functional mutation sites of *CD40* gene. At last, rs4810485 G > T and rs1883832 C > T were identified as the polymorphic loci that were detected in this study (Additional file 1: Figure S1). On the day of admission, 2 mL of peripheral venous blood was extracted from the subjects before 9 AM. Blood samples were anti-coagulated with ethylenediaminetetraacetic acid (EDTA). DNA was extracted using phenol-chloroform extraction method; the concentration was determined and then preserved at -70 °C. DNA fragments of rs4810485 G > T and rs1883832 C > T were amplified with their DNA as the template respectively. Primer was designed with the bio-software Primer Premier 5.0 (Premier, Pala Alto, CA, USA). The upstream primer sequence of rs4810485 G > T was 5’-ATCCCCCAAGTACCTGGCTCCT-3’ and the downstream was 5’-CCTTGCTGCTTCCCTTGCTTTC-3’. The upstream primer sequence of rs1883832 C > T was 5’-CCTCTTCCCCGAAGTCTTCC-3’ and the downstream was 5’-GAAACTCCTGCGCGGTGAAT-3’. The volume of PCR amplification reaction was 20 μL, containing 2.0 μL of 10 × PCR buffer, 2.0 μL of 0.3 mmol/L dNTPs, 1.0 μL of upstream (10 μM) and downstream primers (10 μM) respectively, 1.0 μL of template DNA (2.5 ng/μL), 1.0 U of TaqDNA polymerase. The lack of 20 μL volume could be supplemented by ddH_2_O. Reaction conditions for rs4810485 G > T were a total of 42 cycles of denaturation for 30 s at 94 °C, annealing for 35 s at 56 °C, extending for 45 s at 72 °C, and extending for 10 min at 72 °C as the end of reaction with 320 bp product. PCR amplification product of 10 μL was dealt with by 5 U restriction endonuclease SfaNI in water for 5 h at 37 °C. Reaction conditions for rs1883832 C > T were pre-denatured for 5 min at 94 °C, then a total of 35 cycles of denaturation for 30 s at 94 °C, annealing for 45 s at 61 °C, extending for 45 s at 72 °C, and at last extending for 10 min at 72 °C. PCR amplification product of 10 μL was cleaved at rs1883832 C > T by 5 U restriction endonuclease NcoI, and reaction lasted for 6 h at 37 °C. Shrimp alkaline phosphatase (Promega Corporation, Madison, WI, USA) and exonuclease I (Epicentre) were used to purify PCR product, then SNaPshot Multiplex kit (ABI Company, Oyster Bay, NY, USA) was used for extending reaction, and Promega was used to purify the product of extension. The samples were loaded in ABl3130XL and SNP typing was conducted through GeneMapper4.0 (ABI Company, Oyster Bay, NY, USA).

### Statistical analysis

The statistical analyses were conducted with SPSS21.0, and measurement data were presented by mean ± standard deviation (SD). Data consistent with normal distribution was analyzed using *t*-test and variance analysis, and data not conforming to normal distribution was analyzed using rank-sum test. Enumeration data were presented by number or ratio, differences between groups were analyzed using Chi-Square Test; multiple sets of data were analyzed using partition of chi-square test, and rank sum test was used to compare genotypes of different severities. Binary logistic regression analysis was used to analyze the risk factors related to disease severity of KOA. Disease severity of KOA was taken as the dependent variable, and rs4810485 G > T, rs1883832 C > T, age, gender, BMI, smoking status, alcohol consumption, heavy labor and living environment were taken as the independent variable. Odds ratios (OR), 95% confidential interval (CI), and *P*-value were calculated. *P* < 0.05 was considered statistically significant.

## Results

### Comparisons of baseline characteristics between the KOA group and the control group

There was no significant difference in age, gender, BMI, smoking status, alcohol consumption, and heavy labor between the KOA group and the control group (all *P* > 0.05). The percentage of patients who had lived in a damp and dark environment was much higher in the KOA group than in the control group (89.47% vs. 6.99%). Therefore, living environment was an external factor affecting KOA (Table 1).

**Table 1:** Comparisons of baseline characteristics between the KOA group and the control group

Baseline characteristic	KOA group	Control group	*P*
	(*n* = 133)	(*n* = 143)	
Age (year)	58.24 ± 9.66	56.72 ± 9.43	0.187
Gender (case)			0.214
Male	39	52	
Female	94	91	
BMI (kg/m^2^)	23.60 ± 3.07	22.95 ± 2.82	0.068
Smoking status (%)			
Never	100 (75.19)	102 (71.33)	0.577
Ever	8 (6.02)	7 (4.90)	
Still	25 (18.8)	34 (23.78)	
Alcohol consumption (%)			
Yes	48 (36.09)	48 (33.57)	0.660
No	85 (63.91)	95 (66.43)	
Heavy labor (%)	37 (27.82)	39 (27.27)	0.919
Living environment			<0.001
Good (Bright + dry)	14 (10.53)	133 (93.01)	
Poor (Dark + damp)	119 (89.47)	10 (6.99)	

*Note*: *KOA* Knee osteoarthritis, *BMI* Body mass index

### The DNA sequencing analysis of *CD40* gene

**Fig. 1:**
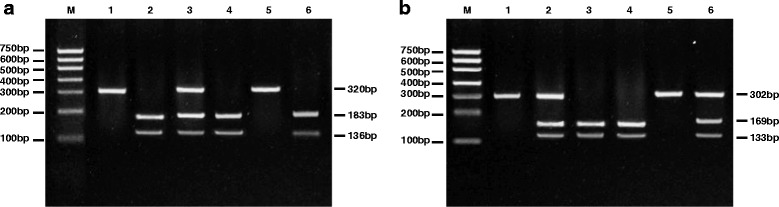
Electrophoresis map of *CD40* rs4810485 G > T and rs1883832 C > T. Note: **a**, *CD40* rs4810485 G > T polymorphism: 1 and 5, GG genotype; 2 and 4, TT genotype; 3 and 6, GT genotype; **b**, *CD40* rs1883832 C > T polymorphism: 1 and 5, TT genotype; 2 and 6, CT genotype; 3 and 4, CC genotype; *CD40*, Cluster of Differentiation 40

Agarose gel electrophoresis of PCR-amplified products showed that rs4810485 G > T was a 320 bp single band before enzyme cleavage. And after enzyme cleavage, G/G homozygote was a 320 bp band (GG genotype); G/T heterozygote included 136 bp, 183 bp, and 320 bp bands (GT genotype); T/T homozygote included 136 bp and 183 bp bands (TT genotype) (Fig. 1a). The results of DNA sequencing were consistent with those tested by PCR-RFLP. PCR-amplified products at rs1883832 C > T were 302 bp. According to the restriction enzyme NcoI fragments, there were three genotypes (Fig. 1b): two bands for CC genotype (169 bp, 133 bp), three bands for CT genotype (302 bp, 169 bp, and 133 bp), and one band for TT genotype (302 bp). Confirmed by DNA sequencing, they were consistent with the results of enzyme digestion.

### Comparisons of genotype frequency distributions and allele frequencies of CD40 rs4810485 G > T and rs1883832 C > T between the KOA and control groups

Genotype distributions and allele frequencies of rs4810485 G > T and rs1883832 C > T in the KOA group and the control group are shown in Table 2. Genotype distributions of all polymorphisms were found to conform to Hardy-Weinberg equilibrium (*P* > 0.05), indicating that the population was well represented. The KOA group was significantly different from the control group in genotype distribution and allele frequency of rs4810485 G > T (*P* < 0.05). Compared with patients carrying GG genotype, patients with TT genotype had significantly lower risk of KOA (GG vs TT: OR = 0.194, 95%CI = 0.097 ~ 0.387, *P <* 0.001). The susceptibility to KOA in individuals carrying T allele significantly decreased compared with those carrying G allele (G vs T: OR = 0.410, 95%CI = 0.290 ~ 0.577, *P* < 0.001), indicating that homozygous TT genotype and T allele may be protective factors for KOA. Besides, patients with rs1883832 C > T TT genotype had increased susceptibility to KOA compared with CC genotype (CC vs TT: OR = 2.914, 95%CI = 1.413 ~ 6.010, *P =* 0.003), which suggests that rs1883832 C > T T allele and mutant homozygous TT genotype increased the susceptibility to KOA.

**Table 2:** Comparisons of genotype distributions and allele frequencies of *CD40* rs4810485 G > T and rs1883832 C > T between the KOA and control groups

SNP	KOA group	Control group	χ2	*P*	OR (95%CI)
rs4810485 G > T					
GG	44 (33.08%)	21 (14.69%)			Ref.
GT	63 (47.37%)	58 (40.56%)	4.226	0.040	0.518 (0.276 ~ 0.974)
TT	26 (19.55%)	64 (44.76%)	22.95	< 0.001	0.194 (0.097 ~ 0.387)
GT + TT	89 (66.92%)	122 (85.31%)	12.95	< 0.001	0.348 (0.194 ~ 0.627)
G allele	151 (57.14%)	100 (34.97%)			Ref.
T allele	115 (42.86%)	186 (65.03%)	26.42	< 0.001	0.410 (0.290 ~ 0.577)
rs1883832 C > T					
CC	21 (15.79%)	43 (30.07%)			Ref.
CT	75 (56.39%)	74 (51.75%)	5.553	0.019	2.075 (1.124 ~ 3.830)
TT	37 (27.82%)	26 (18.18%)	8.595	0.003	2.914 (1.413 ~ 6.010)
CT + TT	96 (72.18%)	117 (81.82%)		0.082	1.680 (0.934 ~ 3.024)
C allele	117 (43.98%)	160 (55.94%)			Refer
T allele	149 (56.02%)	126 (44.06%)	7.884	0.005	1.617 (1.155 ~ 2.264)

*Note*: *CD40* Cluster of Differentiation 40, *KOA* Knee osteoarthritis, *OR* Odds ratio, *CI* Credibility interval, *SNP* Single nucleotide polymorphism

### Correlation analysis between SNPs in *CD40* gene and clinical characteristics of KOA patients

The correlation of rs4810485 G > T and rs1883832 C > T with KOA patients’ age, gender, BMI, smoking status, alcohol consumption, and heavy labor is shown in Table 3. No significant difference was found between different genotypes of rs4810485 G > T and rs1883832 C > T with age, gender, BMI, smoking status, alcohol consumption and heavy labor (all *P* > 0.05).

**Table 3:** Correlations of *CD40* rs4810485 G > T and rs1883832 C > T polymorphisms with clinical characteristics of KOA patients

Clinical characteristic	rs4810485 G > T			*P*	rs1883832 C > T			*P*
	GG (44)	GT (63)	TT (26)		CC (37)	CT (75)	TT (21)	
Age				0.858				0.883
< 61 years	25 (56.82)	39 (61.90)	16 (61.54)		13 (61.90)	46 (61.33)	21 (56.76)	
≥ 61 years	19 (43.18)	24 (38.10)	10 (38.46)		8 (38.10)	29 (38.67)	16 (43.24)	
Gender				0.230				0.300
Male	16 (36.36)	14 (22.22)	9 (34.62)		8 (38.10)	18 (24.00)	13 (35.14)	
Female	28 (63.64)	49 (77.78)	17 (65.38)		13 (61.90)	57 (76.00)	24 (64.86)	
BMI (kg/m^2^)				0.107				0.086
19 ≤ Y ≤ 26	33 (75)	52 (82.54)	16 (61.54)		12 (57.14)	59 (78.67)	30 (81.08)	33 (75)
Y < 19 or Y > 26	11 (25)	11 (17.46)	10 (38.46)		9 (42.86)	16 (21.33)	7 (18.92)	11 (25)
Smoking status (%)				0.699				0.289
Yes	10 (22.7)	11 (17.46)	4 (15.38)		4 (19.05)	11 (14.67)	10 (27.03)	
No	34 (77.3)	52 (82.54)	22 (84.62)		17 (80.95)	64 (85.33)	27 (72.97)	
Alcohol consumption (%)				0.911				0.943
Yes	17 (38.61)	22 (34.92)	9 (34.62)		7 (33.33)	27 (36.00)	14 (37.84)	
No	27 (61.36)	41 (65.08)	17 (65.38)		14 (66.67)	48 (64.00)	23 (62.16)	
Heavy labor	10 (22.73)	19 (30.16)	8 (30.77)	0.653	7 (33.33)	18 (24.00)	12 (32.43)	0.534

*Note*: *CD40* Cluster of Differentiation 40, *KOA* Knee osteoarthritis, *BMI* Body mass index

### The relationship between *CD40* gene polymorphism and KOA severity

The correlation analysis between different genotypes and KOA severity is shown in Table 4. There were significant differences in genotype and allele distributions of rs4810485 G > T and rs1883832 C > T among the mild, moderate and severe groups (all *P* < 0.05). There were more patients with rs4810485 G > T GG genotype and rs1883832 C > T TT genotype in the severe group than other genotypes of these two SNPs.

**Table 4:** The relationship between *CD40* rs4810485 G > T and rs1883832 C > T polymorphisms and the disease severity of KOA

SNP	Severe	Moderate	Mild	χ2	*P*
	(*n* = 40)	(*n* = 44)	(*n* = 49)		
rs4810485 G > T					
GG	26 (63.41)	10 (23.26)	8 (16.33)		
GT	11 (27.5)	24 (54.5)	28 (57.1)	25.38	< 0.001
TT	4 (10.00)	9 (20.5)	13 (26.53)		
G allele	63 (78.75)	44 (50.00)	44 (44.90)		
T allele	19 (23.75)	42 (47.73)	54 (55.10)	20.17	< 0.001
rs1883832 C > T					
CC	3 (7.32)	7 (16.28)	11 (22.4)		
CT	20 (48.78)	25 (58.14)	30 (61.22)	10.11	0.039
TT	18 (43.90)	11 (25.58)	8 (16.33)		
C allele	26 (32.50)	39 (44.32)	52 (53.06)		
T allele	56 (70.00)	47 (53.41)	46 (46.94)	8.358	0.015

*Note*: *CD40* Cluster of Differentiation 40, *KOA* Knee osteoarthritis, *SNP* Single nucleotide polymorphism

### Binary logistic regression analysis for disease severity of KOA

Binary logistic regression analysis was performed and the results showed that rs4810485 G > T and rs1883832 C > T were both related to KOA. TT genotype of rs4810485 G > T could alleviate disease severity of KOA while TT genotype of rs1883832 C > T increases the severity of KOA (both *P* < 0.05). Age, gender, BMI, smoking status, alcohol consumption and heavy labor had little impact on the severity of KOA, while living environment is considered an important external factor that affects KOA severity (all *P* < 0.05) (Table 5).

**Table 5:** Binary logistic regression analysis for the disease severity of KOA

Variables	B	S.E.	Wald	Df	*P*	Exp (B)	95% CI
rs4810485 TT	−1.528	0.612	6.238	1	0.013	0.217	0.065 ~ 0.720
rs1883832 TT	1.333	0.671	3.942	1	0.047	3.793	1.017 ~ 14.140
Age	0.601	0.46	1.703	1	0.192	1.824	0.740 ~ 4.496
Gender	0.439	0.494	0.789	1	0.374	1.551	0.589 ~ 4.085
BMI	0.421	0.595	0.499	1	0.480	1.523	0.474 ~ 4.892
Smoking status	0.295	0.278	1.125	1	0.289	1.343	0.779 ~ 2.317
Alcohol consumption	−0.061	0.491	0.015	1	0.901	0.941	0.359 ~ 2.465
Heavy labor	−0.006	0.526	0	1	0.991	0.994	0.355 ~ 2.788
Living environment	−3.491	1.13	9.538	1	0.002	0.030	0.003 ~ 0.279

*Note*: *KOA* Knee osteoarthritis, *BMI* Body mass index, *B* Beta, *S.E.* Standard error, *CI* Credibility interval

## Discussion

Osteoarthritis is the world’s most common inflammatory joint disease and one of the main causes of disability [24]. KOA is a kind of osteoarthritis that greatly impacts the life quality of patients [25]. KOA is a main cause of constant knee pain. According to statistics, majority of people suffer from frequent knee pain, which leads to the limitation of joint function and activity [26]. In recent years, with people’s deepening awareness about the impact of genetic factors on the KOA onset, the role of gene polymorphism in the occurrence and development of KOA has attracted widespread attention [27]. This paper intends to explore the correlation of gene polymorphism of *CD40* rs4810485 G > T and rs1883832 C > T with disease susceptibility and severity in KOA.

First, this study found that, patients who had lived in damp and dark environments had a higher risk of suffering from KOA, suggesting that living environment is an external factor, which was related with the severity of KOA. A previous study has confirmed damp living environment as one of the risk factors for KOA [28]. Therefore, improvement of ventilation and lighting of a living environment can also effectively alleviate susceptibility to KOA [29].

The study found that homozygous TT genotype and T allele of rs4810485 G > T may be protective factors in the pathogenesis of KOA, while T allele and homozygote TT genotype of rs1883832 C > T increased the susceptibility to KOA. Besides, there were more patients with rs4810485 G > T GG genotype and rs1883832 C > T TT genotype in the severe group than other genotypes of these two SNPs. Our binary logistic regression analysis verified that rs4810485 G > T TT genotype decreased disease severity of KOA, while rs1883832 C > T TT genotype increased disease severity of KOA. At present, *CD40* rs4810485 is one of the most studied gene polymorphisms that is closely related to the susceptibility to rheumatoid arthritis (RA) and systemic lupus erythematosus (SLE) [30, 31]. *CD40*, rs1883832 polymorphism can produce high expression of *CD40* by up-regulating the transcription or translation efficiency of *CD40* gene, and the abnormal expression of *CD40* will lead to the increase of pro-inflammatory cytokine and cause diseases [16, 32]. A recent study in Spanish postmenopausal women pointed out that patients with T allele and TT genotype of *CD40* rs1883832 C > T have decreased CD40 expression and lowered bone mineral density (BMD) at femoral neck and spine sites, leading to the reduced expression of osteoprotegerin (OPG), thereby increasing the susceptibility to osteopenia or osteoporosis [33]. Previous evidences showed that KOA patients also have greater BMD and higher OPG levels in the serum and synovial fluid than the normal controls [34, 35]. In addition, shortage of *CD40* rs4810485 T in RA and SLE is due to decreased expression of *CD40* in peripheral blood mononuclear cells and B cells, while sustained expression of CD40L and elevated expression of *CD40* in RA and SLE patients are the main causes of enhanced activation of humoral and cellular immunity, activation of non-cellular immune target, and eventually disease [36]. It has been found that in ACS and breast cancer patients, the frequency of CC genotype in *CD40-1* T > C increased significantly compared to healthy controls, and *CD40-1* T > C significantly increased the risk of these two diseases [37].

However it should be disclosed that the mechanism of correlation is not fully understood at present. Although 133 KOA patients and 143 healthy controls were enrolled in this study, the sample size was still not big enough, and multiple risk factors were revealed for KOA in previous researches that we failed to take into considerations due to limited sample and funding, such as meniscectomy and regular sports participation [38, 39]. Besides, the association between a high CD40 expression and patients with TT genotype of rs1883832 C > T requires further investigation. In addition, replication and fine mapping of SNP in KOA patients are expected for further research.

## Conclusions

In summary, our results demonstrated that BMI and living environment are external factors for the susceptibility to KOA. TT genotype and T allele of rs4810485 G > T are protective factors for KOA, while C allele and genotype CC of rs1883832 C > T can increase the susceptibility of KOA. The results of this study opened a new avenue for investigation that may lend insight into KOA.

## Additional file

Additional file 1: Figure S1. Block diagram of *CD40* rs4810485 G > T and rs1883832 C > T. Note: *CD40*, Cluster of Differentiation 40. (EPS 928 kb)

## Abbreviations

ACL: Anterior cruciate ligament
ACS: Acute coronary syndrome
ARHP: Association of Rheumatology Health Professionals
BMD: Bone mineral density
BMI: Body mass index
CD40: Cluster of Differentiation 40
EDTA: Ethylenediaminetetraacetic acid
GCA: Giant cell arteritis
KL: Kellgren-Lawrence
KOA: Knee osteoarthritis
OPG: Osteoprotegerin
OR: Odds ratios
PCR-RFLP: Polymerase chain reaction–restriction fragment length polymorphism
RA: Rheumatoid arthritis
SD: Standard deviation
SLE: Systemic lupus erythematosus
SLE: Systemic lupus erythematosus
SNPs: Single nucleotide polymorphisms.

## References

[1] Kirkley A, Birmingham TB, Litchfield RB, Giffin JR, Willits KR, Wong CJ, Feagan BG, Donner A, Griffin SH, D’Ascanio LM, Pope JE, Fowler PJ (2008). A randomized trial of arthroscopic surgery for osteoarthritis of the knee. N Engl J Med.

[2] Blagojevic M, Jinks C, Jeffery A, Jordan KP (2010). Risk factors for onset of osteoarthritis of the knee in older adults: a systematic review and meta-analysis. Osteoarthritis Cartilage.

[3] Zhang W, Nuki G, Moskowitz RW, Abramson S, Altman RD, Arden NK, Bierma-Zeinstra S, Brandt KD, Croft P, Doherty M, Dougados M, Hochberg M, Hunter DJ, Kwoh K, Lohmander LS, Tugwell P (2010). OARSI recommendations for the management of hip and knee osteoarthritis: part III: Changes in evidence following systematic cumulative update of research published through January 2009. Osteoarthritis Cartilage.

[4] Williams A, Qian Y, Golla S, Chu CR (2012). UTE-T2 * mapping detects sub-clinical meniscus injury after anterior cruciate ligament tear. Osteoarthritis Cartilage.

[5] Oiestad BE, Engebretsen L, Storheim K, Risberg MA (2009). Knee osteoarthritis after anterior cruciate ligament injury: a systematic review. Am J Sports Med.

[6] Hunter DJ, Niu JB, Zhang Y, LaValley M, McLennan CE, Hudelmaier M, Eckstein F, Felson DT (2008). Premorbid knee osteoarthritis is not characterised by diffuse thinness: the Framingham Osteoarthritis Study. Ann Rheum Dis.

[7] Morio T, Hanissian S, Geha RS (1995). Characterization of a 23-kDa protein associated with CD40. Proc Natl Acad Sci U S A.

[8] Georgakis GV, Younes A (2005). Cytokines and lymphomas. Cancer Treat Res.

[9] Park CI, Hirono I, Hwang JY, Aoki T (2005). Characterization and expression of a CD40 homolog gene in Japanese flounder Paralichthys olivaceus. Immunogenetics.

[10] Sokolova EA, Malkova NA, Korobko DS, Rozhdestvenskii AS, Kakulya AV, Khanokh EV, Delov RA, Platonov FA, Popova TY, EG A’ e, Zagorskaya NN, Alifirova VM, Titova MA, Smagina IV, SA E’ c, Popovtseva AV, Puzyrev VP, Kulakova OG, Tsareva EY, Favorova OO, Shchur SG, Lashch NY, Popova NF, Popova EV, Gusev EI, Boyko AN, Aulchenko YS, Filipenko ML (2013). Association of SNPs of CD40 gene with multiple sclerosis in Russians. PLoS One.

[11] Pasterkamp G, de Kleijn D (2008). Microparticles, debris that hurts. J Am Coll Cardiol.

[12] Tian C, Qin W, Li L, Zheng W, Qiu F (2010). A common polymorphism in CD40 Kozak sequence (-1C/T) is associated with acute coronary syndrome. Biomed Pharmacother.

[13] Jacobson EM, Huber AK, Akeno N, Sivak M, Li CW, Concepcion E, Ho K, Tomer Y (2007). A CD40 Kozak sequence polymorphism and susceptibility to antibody-mediated autoimmune conditions: the role of CD40 tissue-specific expression. Genes Immun.

[14] Wang M, Li Y, Li W, Xia ZE, Wu Q (2011). The CD40 gene polymorphism rs1883832 is associated with risk of acute coronary syndrome in a Chinese case-control study. DNA Cell Biol.

[15] Sokolove J, Lepus CM (2013). Role of inflammation in the pathogenesis of osteoarthritis: latest findings and interpretations. Ther Adv Musculoskelet Dis.

[16] Rodriguez-Rodriguez L, Castaneda S, Vazquez-Rodriguez TR, Morado IC, Mari-Alfonso B, Gomez-Vaquero C, Miranda-Filloy JA, Narvaez J, Ortego-Centeno N, Blanco R, Fernandez-Gutierrez B, Martin J, Gonzalez-Gay MA (2010). Influence of CD40 rs1883832 polymorphism in susceptibility to and clinical manifestations of biopsy-proven giant cell arteritis. J Rheumatol.

[17] Chen JM, Guo J, Wei CD, Wang CF, Luo HC, Wei YS, Lan Y (2015). The association of CD40 polymorphisms with CD40 serum levels and risk of systemic lupus erythematosus. BMC Genet.

[18] Piotrowski P, Lianeri M, Wudarski M, Olesinska M, Jagodzinski PP (2013). Single nucleotide polymorphism of CD40 region and the risk of systemic lupus erythematosus. Lupus.

[19] Vazgiourakis VM, Zervou MI, Choulaki C, Bertsias G, Melissourgaki M, Yilmaz N, Sidiropoulos P, Plant D, Trouw LA, Toes RE, Kardassis D, Yavuz S, Boumpas DT, Goulielmos GN (2011). A common SNP in the CD40 region is associated with systemic lupus erythematosus and correlates with altered CD40 expression: implications for the pathogenesis. Ann Rheum Dis.

[20] Celli BR, MacNee W, Force AET (2004). Standards for the diagnosis and treatment of patients with COPD: a summary of the ATS/ERS position paper. Eur Respir J.

[21] Ou GP, Xiao J, Zheng ZY, et al. A survey on risk factors for elderly knee osteoarthritis. Chin J Tissue Eng Res. 2012;16(50):9463–70.

[22] Monfort J, Nacher M, Montell E, Vila J, Verges J, Benito P (2005). Chondroitin sulfate and hyaluronic acid (500-730 kda) inhibit stromelysin-1 synthesis in human osteoarthritic chondrocytes. Drugs Exp Clin Res.

[23] Yuan HY, Chiou JJ, Tseng WH, Liu CH, Liu CK, Lin YJ, Wang HH, Yao A, Chen YT, Hsu CN (2006). FASTSNP: an always up-to-date and extendable service for SNP function analysis and prioritization. Nucleic Acids Res.

[24] Strand V, Lim S, Takamura J (2016). Evidence for safety of retreatment with a single intra-articular injection of Gel-200 for treatment of osteoarthritis of the knee from the double-blind pivotal and open-label retreatment clinical trials. BMC Musculoskelet Disord.

[25] Shakoor N, Lidtke RH, Wimmer MA, Mikolaitis RA, Foucher KC, Thorp LE, Fogg LF, Block JA (2013). Improvement in knee loading after use of specialized footwear for knee osteoarthritis: results of a six-month pilot investigation. Arthritis Rheum.

[26] Turkiewicz A, Gerhardsson de Verdier M, Engstrom G, Nilsson PM, Mellstrom C, Lohmander LS, Englund M (2015). Prevalence of knee pain and knee OA in southern Sweden and the proportion that seeks medical care. Rheumatology (Oxford).

[27] Valdes AM, Spector TD (2011). Genetic epidemiology of hip and knee osteoarthritis. Nat Rev Rheumatol.

[28] McAlindon T, Formica M, Schmid CH, Fletcher J (2007). Changes in barometric pressure and ambient temperature influence osteoarthritis pain. Am J Med.

[29] Zhang J, Song L, Liu G, Zhang A, Dong H, Liu Z, Li X, Luo J (2013). Risk factors for and prevalence of knee osteoarthritis in the rural areas of Shanxi Province, North China: a COPCORD study. Rheumatol Int.

[30] Raychaudhuri S, Remmers EF, Lee AT, Hackett R, Guiducci C, Burtt NP, Gianniny L, Korman BD, Padyukov L, Kurreeman FA, Chang M, Catanese JJ, Ding B, Wong S, van der Helm-van Mil AH, Neale BM, Coblyn J, Cui J, Tak PP, Wolbink GJ, Crusius JB, van der Horst-Bruinsma IE, Criswell LA, Amos CI, Seldin MF, Kastner DL, Ardlie KG, Alfredsson L, Costenbader KH, Altshuler D, Huizinga TW, Shadick NA, Weinblatt ME, de Vries N, Worthington J, Seielstad M, Toes RE, Karlson EW, Begovich AB, Klareskog L, Gregersen PK, Daly MJ, Plenge RM (2008). Common variants at CD40 and other loci confer risk of rheumatoid arthritis. Nat Genet.

[31] Joo YB, Park BL, Shin HD, Park SY, Kim I, Bae SC (2013). Association of genetic polymorphisms in CD40 with susceptibility to SLE in the Korean population. Rheumatology (Oxford).

[32] Lee YC, Kuo HC, Chang JS, Chang LY, Huang LM, Chen MR, Liang CD, Chi H, Huang FY, Lee ML, Huang YC, Hwang B, Chiu NC, Hwang KP, Lee PC, Chang LC, Liu YM, Chen YJ, Chen CH, Taiwan Pediatric IDA, Chen YT, Tsai FJ, Wu JY (2012). Two new susceptibility loci for Kawasaki disease identified through genome-wide association analysis. Nat Genet.

[33] Pineda B, Laporta P, Hermenegildo C, Cano A, Garcia-Perez MA (2008). A C > T polymorphism located at position -1 of the Kozak sequence of CD40 gene is associated with low bone mass in Spanish postmenopausal women. Osteoporos Int.

[34] Sowers M, Lachance L, Jamadar D, Hochberg MC, Hollis B, Crutchfield M, Jannausch ML (1999). The associations of bone mineral density and bone turnover markers with osteoarthritis of the hand and knee in pre- and perimenopausal women. Arthritis Rheum.

[35] Pilichou A, Papassotiriou I, Michalakakou K, Fessatou S, Fandridis E, Papachristou G, Terpos E (2008). High levels of synovial fluid osteoprotegerin (OPG) and increased serum ratio of receptor activator of nuclear factor-kappa B ligand (RANKL) to OPG correlate with disease severity in patients with primary knee osteoarthritis. Clin Biochem.

[36] Lee YH, Bae SC, Choi SJ, Ji JD, Song GG (2015). Associations between the functional CD40 rs4810485 G/T polymorphism and susceptibility to rheumatoid arthritis and systemic lupus erythematosus: a meta-analysis. Lupus.

[37] Dolen Y, Yilmaz G, Esendagli G, Guler NE, Guc D (2010). CD40–1C > T single nucleotide polymorphism and CD40 expression on breast tumors. Cytokine.

[38] Englund M, Lohmander LS (2004). Risk factors for symptomatic knee osteoarthritis fifteen to twenty-two years after meniscectomy. Arthritis Rheum.

[39] Cooper C, Snow S, McAlindon TE, Kellingray S, Stuart B, Coggon D, Dieppe PA (2000). Risk factors for the incidence and progression of radiographic knee osteoarthritis. Arthritis Rheum.

